# A Change of Heart: Case Series of Peripartum Cardiomyopathy

**DOI:** 10.1155/2013/563158

**Published:** 2013-12-22

**Authors:** Sean Martin, Daniel Short, Chih Mun Wong, Dina McLellan

**Affiliations:** ^1^Maternity Unit, Wishaw General Hospital, 50 Netherton Street, Wishaw, South Lanarkshire ML2 0DP, UK; ^2^Scottish Advanced Heart Failure Service, Golden Jubilee National Hospital, Clydebank, Glasgow G42 9TY, UK

## Abstract

Peripartum cardiomyopathy (PPCM) is an uncommon disease of pregnancy, occurring in about 1 in 2000 live births, and is characterized by the development of heart failure, due to left ventricular systolic dysfunction. It is associated with high rates of maternal and neonatal mortality. Cardiac disease is the leading cause of maternal death in the UK: PPCM accounts for about 17% of these. Clinical findings of decompensated heart failure (HF) are often masked by the normal physiological changes seen in pregnancy making the diagnosis challenging. A high index of suspicion is essential—prompting referral for echocardiogram, which is crucial for diagnosis. Favourable prognosis is dependent on the early initiation of HF medications. Although full recovery occurs in around half of cases, left ventricular systolic dysfunction persists in a significant proportion of patients with PPCM and the risk of recurrence in subsequent pregnancies is high. The pathophysiology of PPCM is under intense research. We present four patients with PPCM and a review of the literature. Owing to the diagnostic challenge of PPCM and decompensated HF in pregnant mothers and its high mortality rate without treatment, prompt investigation and referral are key to improving maternal survival.

## 1. Introduction

Cardiac disease is the leading cause of maternal death in the UK [1]. Peripartum cardiomyopathy (PPCM) is an idiopathic cardiomyopathy that presents in the puerperium as heart failure secondary to left ventricular systolic dysfunction in the absence of another cause of heart failure [2]. The incidence of PPCM is around 1 in 2000 live births [3] and accounts for about 17% of the deaths secondary to cardiac disease in the UK [1].

We present a series of four women who presented with PPCM in our centre, of which two died. Their clinical presentations were variable and, so, represented an important diagnostic challenge.

## 2. Case Presentation

### 2.1. Case One

**Figure 1:**
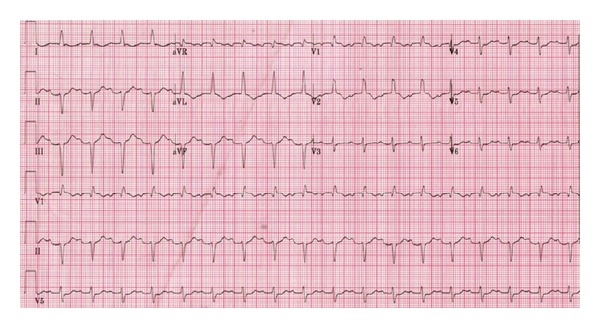
ECG on presentation (case one). Showing sinus tachycardia, left axis deviation, and T-wave changes.

**Figure 2:**
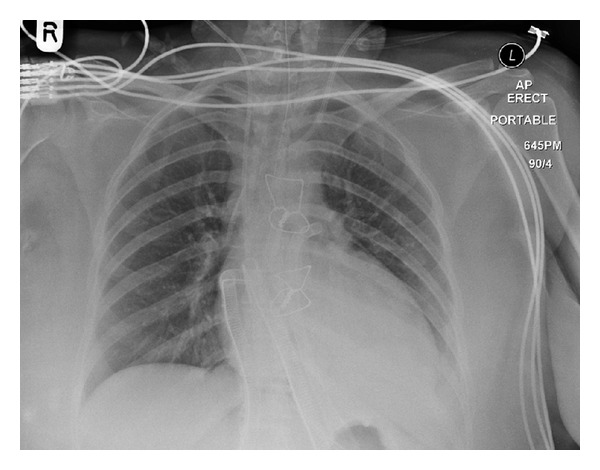
Chest X-ray (case one). Showing implanted BiVAD.

A 26-year-old woman gave birth by spontaneous vaginal delivery at 41 weeks to her third child. She presented to hospital 6 months later with dyspnoea, light-headedness, and palpitations. ECG on admission showed sinus tachycardia, left axis deviation, and T-wave changes (Figure 1). Chest X-ray showed pulmonary oedema. Her rapid deterioration led to her transfer to the National Heart Failure Unit, where she had a short-term biventricular assist device (BiVAD, Figure 2) implanted. She suffered two on-table cardiac arrests, requiring manual cardiac massage, while the BiVAD was being implanted.

Unfortunately, her postoperative period was complicated by acute ischaemia of the left lower limb requiring above knee amputation and a prolonged period of renal replacement therapy. Her cardiac function improved over a period of two months and the BiVAD was successfully explanted. She had, remarkably, good cardiac function after explant. She remains well to date on beta-blocker and ACE-inhibitor.

### 2.2. Case Two

A 25-year-old primiparous woman with a history of insulin-dependent diabetes mellitus presented at 34 weeks' gestation with preeclampsia, for which she was given steroids. Emergency Caesarean section was undertaken at 35 weeks for preeclamptic toxaemia, following which she was discharged home on day 4. She was readmitted 2 days later with dyspnoea and orthopnoea. Initial differential diagnoses were panic attack and pulmonary thromboembolism. The diagnosis of decompensated HF was only made following a chest X-ray showing pulmonary oedema.

She was intubated and transferred to the National Heart Failure Unit. She was supported by an intra-aortic balloon pump, which was successfully weaned after 5 days. Her follow up echocardiogram showed good ventricular functions. She remains well to date on beta-blocker, ACE-inhibitor, and aldosterone antagonist. She also had a trial of bromocriptine for three months.

### 2.3. Case Three

A 31-year-old primiparous woman presented a 17 weeks' gestation with dyspnoea, which had worsened by 22-week review. At 29 weeks, she represented with a 3-week history of palpitations, tachycardia, and worsening dyspnoea and was discharged home following a normal ECG. She subsequently deteriorated, with further worsening dyspnoea, and was treated at primary care with antibiotics, steroids, and inhalers. She was, again, readmitted at 32 + 6 weeks with dyspnoea at rest and tachycardia.

Cardiomyopathy was diagnosed, she had an emergency Caesarean section and delivered a live baby, and then she was transferred to a cardiac unit. Cardiac function further deteriorated, and she died in the postnatal period.

### 2.4. Case Four

A 35-year-old woman, with one previous pregnancy, gave birth by elective Caesarean section at 39 weeks to a live male. She had previously collapsed at home at 28 weeks' gestation. She died in the postnatal period, and cardiomyopathy was demonstrated at postmortem.

## 3. Discussion

Peripartum cardiomyopathy (PPCM) is a rare condition, with an incidence estimated to be around 1 in 2000 live births, and is associated with high rates of maternal and neonatal morbidity and mortality [3].

It is characterized by the deterioration of left ventricular systolic function towards the end of pregnancy, or in the postnatal period in the absence of another cause for heart failure [2]. Recurrence is common in subsequent pregnancy [4], and a genetic basis for the disease has been posited [5, 6] and so has implications for family planning.

The aetiology of PPCM is currently unclear, though this is currently the subject of much promising research, which may pave the way for novel approaches to treatment, which will be discussed later. It may well be that some cases are hitherto undiagnosed cardiomyopathies that only become overt in pregnancy; it is important that it is borne in mind that cardiac disease may come to the fore during gestation and in the peripartum period.

### 3.1. Diagnosis

Diagnosis is mainly based on clinical suspicion and echocardiogram. However, a problem lies in that many of the signs and symptoms of normal pregnancy are indistinguishable from heart failure, and the condition may be missed completely or misdiagnosed. Electrocardiogram (ECG) may be relatively unremarkable (Figure 1), and chest X-ray may have few of the classical signs of decompensated heart failure, falsely reassuring attending physicians, leading to delay in diagnosis.

The Centre for Maternal and Child Enquiries has thus suggested that “women in late pregnancy or within 6 months of delivery with symptoms of breathlessness, or oedema, or orthopnoea and the signs of tachypnoea and tachycardia may have PPCM, and investigation with a chest X-ray and echocardiogram are indicated” [1].

There are recognised risk factors for PPCM, which provide us with a tool to identify those who are at risk of developing the condition. Obesity, smoking, excessive alcohol consumption, and malnutrition are all potentially modifiable risk factors for the disease, and education about these risk factors should inform all patients' antenatal period regardless of any particular suspicion of PPCM. Any previous personal or family history of heart disease should alert clinicians to a high-risk pregnancy.

Several recommendations for alterations to service provision in order to improve maternal outcomes were made by the “Saving Mothers' Lives” report [1]. Prepregnancy counseling should be offered, starting with those women who have preexisting medical illnesses, particularly those women with congenital or known acquired cardiac disease and those with obesity—both significant risk factors for the development of PPCM. Immediate referral to specialist care is required when symptoms first occur. However, we have seen from our case series that the presenting symptoms of PPCM may be easily mistaken for normal physiological response to pregnancy or chest infection. Another recommendation which is directly applicable to PPCM, is the need for specialised maternal pathology services, including a reduction in the number of facilities that conduct peripartum postmortem examinations. In our series, one case of PPCM was only diagnosed at postmortem, thus highlighting the importance of examination by an experienced cardiac pathologist who has access to both clinical information and family history.

### 3.2. Management

The exact management strategy for PPCM depends on the individual clinical case but is essentially based on the stability of the patient. Acute heart failure and stable heart failure are managed quite differently. Acute heart failure in the peripartum period is managed [2] in just the same way as acute heart failure at any other time of life, with pulmonary oedema and hypoxia treated rapidly with oxygen, noninvasive ventilation if required, and diuretics or nitrates in the volume overloaded. Inotropic support should be used in those with evidence of shock. For patients who continue to deteriorate despite optimal medical management, mechanical circulatory support such as a ventricular assist device (VAD) should be considered. The patients can be supported by either a short- or long-term VAD until recovery of ventricular function has been established.

Management strategies for those with stable heart failure in PPCM [2] are dictated by whether the patient is postpartum or still pregnant, with consideration being given to the acceptability of the drugs used to treat heart failure during pregnancy. The drugs used are largely the same as those used for any other form of heart failure. ACE-inhibitors and angiotensin receptor blockers are toxic to the fetus and should be avoided in pregnancy, as should certain diuretics due to the possibility of reduced blood flow to the placenta. Modifications can be made to current regimes to make drugs choices and combinations safe and effective during pregnancy; in particular, beta-blockers have not been proven to be teratogenic [2]. Caution should be exercised when considering drugs for which there is limited data available in pregnancy.

### 3.3. Prognosis

More than 50% of women recover without complication, with left ventricular systolic function at rest returning to normal [4]. The risk of recurrence in subsequent pregnancy is high, especially if left ventricular function has not returned to normal, and is usually more serious in these women [4]. Some reduction in left ventricular ejection fraction seems inevitable in all patients, though it may be subclinical.

### 3.4. Novel Approaches

#### 3.4.1. As a Genetic Disorder

Recent genome-wide and family studies have shown a possible basis for the aetiology of PPCM and may present a novel screening tool for at risk populations. It has been shown to show some concordance in families, though this may simply be a first manifestation of familial dilated cardiomyopathy [5]. A genome-wide study has shown a strong association between a gene on chromosome 12 and PPCM [6].

#### 3.4.2. As a Disorder of Prolactin Cleavage

It has been shown in mice that a product of protein cleavage causes impairment of cardiac myocyte function resulting in PPCM due to its antiangiogenic and proapoptotic properties. This effect was successfully abrogated in these mice by administration of bromocriptine and by inhibiting prolactin secretion and may hold therapeutic promise in humans [7].

#### 3.4.3. As a Disorder of Cytokine Imbalance

Inflammatory markers including TNF-*α*, IFN-*Υ*, IL-6, and CRP have been shown to be higher in PPCM than in healthy controls [8], though it is unclear if raised levels of these mediators are causative or reactive. Restoration of cytokine balance may, therefore, be an area that holds some promise for therapy.

#### 3.4.4. As a Disordered Immune Response

Antibodies directed against cardiac tissue have been found in PPCM patients [9, 10], though it remains unclear whether or not autoimmune disease is causative or if the antibodies are raised when the epitopes are unmasked following cardiac myocyte damage of another mechanism.

## 4. Conclusion

Due to its rarity, variability in presentation, and potential for mortality, PPCM should be considered in women that present with features consistent with left ventricular failure, though it should be borne in mind that this can mimic the normal physiological changes of pregnancy. And when it does occur, careful cardiac followup is essential.
